# Correlation between brain function and ADHD symptom changes in children with ADHD following a few-foods diet: an open-label intervention trial

**DOI:** 10.1038/s41598-021-01684-7

**Published:** 2021-11-12

**Authors:** Saartje Hontelez, Tim Stobernack, Lidy M. Pelsser, Peter van Baarlen, Klaas Frankena, Martine M. Groefsema, Michiel Kleerebezem, Rob Rodrigues Pereira, Elbrich M. Postma, Paul A. M. Smeets, Marion A. Stopyra, Marcel P. Zwiers, Esther Aarts

**Affiliations:** 1 Host-Microbe Interactomics, Wageningen University and Research, De Elst 1, 6708 WD Wageningen, The Netherlands; 2 ADHD Research Centre, Eindhoven, The Netherlands; 3 Adaptation Physiology, Wageningen University and Research, Wageningen, The Netherlands; 4 Behavioural Science Institute, Radboud University, Nijmegen, The Netherlands; 5 Medical Centre Kinderplein, Rotterdam, The Netherlands; 6 Division of Human Nutrition and Health, Wageningen University and Research, Wageningen, The Netherlands; 7 Department of General Internal Medicine and Psychosomatics, University Hospital Heidelberg, Heidelberg, Germany; 8 Donders Institute for Brain, Cognition and Behaviour, Centre for Cognitive Neuroimaging, Radboud University, Nijmegen, The Netherlands

**Keywords:** Paediatric research, Adaptive clinical trial, Nutrition, ADHD

## Abstract

Research into the effect of nutrition on attention-deficit hyperactivity disorder (ADHD) in children has shown that the few-foods diet (FFD) substantially decreases ADHD symptoms in 60% of children. However, the underlying mechanism is unknown. In this open-label nutritional intervention study we investigated whether behavioural changes after following an FFD are associated with changes in brain function during inhibitory control in 79 boys with ADHD, aged 8–10 years. Parents completed the ADHD Rating Scale before (t1) and after the FFD (t2). Functional magnetic resonance imaging (fMRI) scans were acquired during a stop-signal task at t1 and t2, and initial subject-level analyses were done blinded for ARS scores. Fifty (63%) participants were diet responders, showing a decrease of ADHD symptoms of at least 40%. Fifty-three children had fMRI scans of sufficient quality for further analysis. Region-of-interest analyses demonstrated that brain activation in regions implicated in the stop-signal task was not associated with ADHD symptom change. However, whole-brain analyses revealed a correlation between ADHD symptom decrease and increased precuneus activation (p_FWE(cluster)_ = 0.015 for StopSuccess > Go trials and p_FWE(cluster)_ < 0.001 for StopSuccess > StopFail trials). These results provide evidence for a neurocognitive mechanism underlying the efficacy of a few-foods diet in children with ADHD.

## Introduction

Attention-deficit hyperactivity disorder (ADHD) is a neurodevelopmental disorder characterized by inattentiveness, hyperactivity and impulsiveness, and often co-occurs with other psychiatric disorders such as oppositional defiant disorder (ODD)^1^. Generally prescribed drugs for ADHD are not effective 24 h per day and can cause sleeping problems, decreased appetite, headache and stomach-ache^2^, frequently resulting in discontinuation of medication^3^. Therefore, novel treatments, preferably aiming at the underlying triggers or causes of ADHD, are needed.

Dietary interventions can modulate behaviour and mental health^4^. Supplementation or restriction of some nutrients or foods, have generally shown small effects in relieving ADHD symptoms^5,6^. However, meta-analyses of double-blind placebo-controlled (DBPC) diet restriction studies have reported moderate to large effect sizes (SSMD = 0.8; *p* = 0.04^7^ and SMD = 0.51; *p* = 0.06^8^ when applying a modified few-foods diet (FFD)^7,8^. A systematic review of these meta-analyses showed that inclusion of researcher’s observation ratings rather than teacher ratings in the meta-analysis of Sonuga-Barke et al*.* (2013)^8^—according to Sonuga-Barke et al*.* (2013)^8^ observer ratings would be ‘better probably blinded’ than teacher ratings—resulted in an overall SMD of 0.57; *p* = 0.024^6^. Based on the evidence provided by the DBPC studies, the FFD was included in an ADHD treatment protocol^9^.

To assure the blinded design, the DBPC studies could not apply the most stringent FFD, in which only a few foods are allowed^6^. Subsequent open or single-blinded randomized controlled trials did apply an optimal FFD showing large effects (ES = 1.26–2.35)^10–12^, while a recent uncontrolled FFD study in children with ADHD showed significant behavioural improvements according to blinded video ratings (ES = 1.42) as well as to non-blinded assessments (ES = 1.54)^13^. Yorgidis et al.^14^ stated that research has shown that ‘nutrition is a strong mediator and/or moderator of ADHD symptoms’. However, the impact of the FFD on ADHD is still disputed.

Knowledge about the mechanism underlying the effect of an FFD on ADHD may provide a better understanding of the association between diet and ADHD. Since ADHD has been associated with impaired cognitive control processes in the brain, such as response inhibition and interference inhibition^15^, we aimed to investigate whether behavioural changes after following an FFD are associated with changes in brain activation during inhibition tasks in children with ADHD.

Brain activation in response inhibition (stop-signal and Go/No-Go tasks) and interference inhibition (Stroop and Flanker tasks) can be assessed by blood oxygen level dependent (BOLD) changes in functional magnetic resonance imaging (fMRI). Children with ADHD have decreased activation in various brain regions during inhibition, including the prefrontal cortex, putamen, thalamus, precuneus and parietal and temporal lobes^15^. Psychostimulants have been shown to increase task-related activation in various brain regions in children with ADHD^15,16^. The impact of the FFD on brain activation in children with ADHD has never been investigated, however, some nutritional studies have been performed^17–20^, one of which first used an FFD approach to define which foods the participating children with ADHD were susceptible to, and subsequently showed an increased activity in frontotemporal areas after the children consumed ADHD provoking foods^18^.

Our primary objective was to investigate whether ADHD symptom changes following an FFD are reflected in neural activation changes in the brain. To assess brain activation, fMRI measurements of BOLD responses during response inhibition (stop-signal task) and interference inhibition (Flanker task) were compared before (t1) and after (t2) a 5-week FFD intervention^21^. We hypothesized that the observed changes in ADHD symptoms after the intervention are associated with changes in activation of brain regions that are involved in task execution, using task-related regions-of-interest (ROIs). For our secondary objective we employed a whole-brain approach, investigating the changes in activation for other regions that were not expected a priori.

## Results

**Figure 1:**
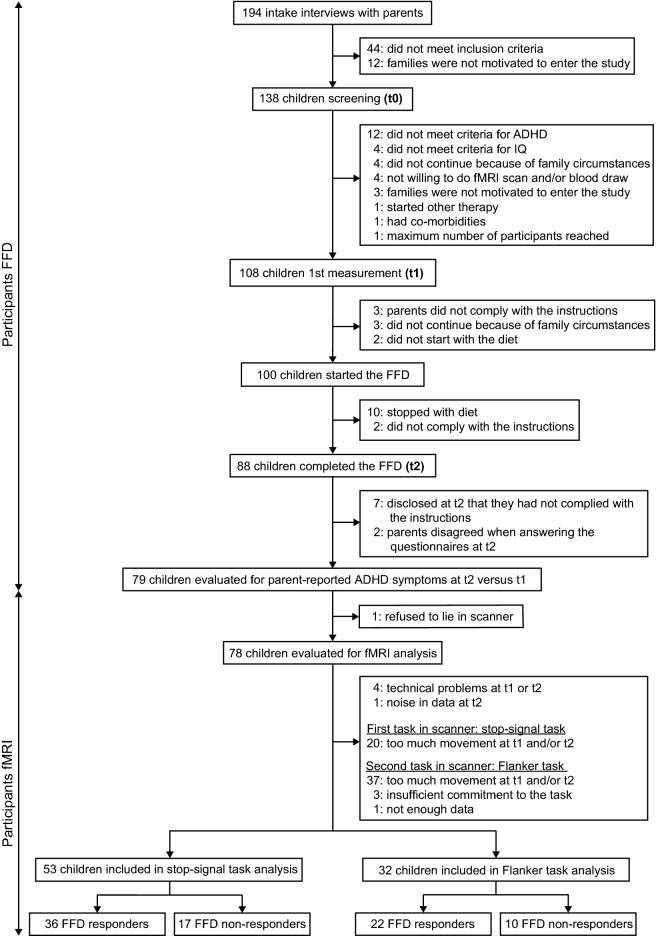
Flowchart participants FFD and fMRI. *ADHD* attention-deficit hyperactivity disorder, *FFD* few-foods diet, *IQ* intelligence quotient, *Non-resp* non-responder (< 40% ARS score decrease at t2 compared to t1), *Resp* responder (≥ 40% ARS score decrease at t2 compared to t1).

Between February 19, 2018, and April 30, 2019, 138 children were screened (t0) for participation in the BRAIN study. Of the 100 children that started with the FFD, 12 stopped the diet prematurely and nine were excluded from analysis. Of the remaining 79 children, 53 were included for the stop-signal task and 32 for the Flanker task analyses (Fig. 1). Sixty-eight of 79 children followed the most restricted FFD; 11 children were kept on the extended FFD, because their parents reported major behavioural improvements of 72–98% while following the extended FFD. Baseline characteristics are provided in Table 1.

**Table 1:** Baseline characteristics of all participants (n = 79)^a^ at t1 and their significance with ARS score decrease (%).

Characteristic	Distributions	*p* value
Age, mean (SD)	9.21 (0.85)	0.07^b^
IQ, mean (SD)	108.08 (12.34)	0.92^b^
**ADHD type, n (%)**		0.83^c^
Combined	70 (89)	
Inattentive	5 (6)	
Hyperactive/impulsive	4 (5)	
**ADHD diagnosis, n (%)**^d^		0.94^c^
ADHD diagnosis prior to start study	53 (67)	
No ADHD diagnosis prior to start study	26 (33)	
**ODD comorbidity**		0.83^c^
Present	58 (73)	
Absent	21 (27)	
**Medication, n (%)**		0.015^c^
Never used ADHD medication	43 (54)	
Has used ADHD medication	36 (46)	

*ADHD* attention-deficit hyperactivity disorder, *ARS* attention-deficit hyperactivity disorder (ADHD) rating scale, *FFD* few-foods diet, *ODD* oppositional defiant disorder, *IQ* intelligence quotient, *SD* standard deviation, *t1* before start FFD.

^a^Also for the subgroups included in the fMRI analyses (n = 53 for the stop-signal task and n = 32 for the Flanker task), p-values indicated no significant association between baseline characteristics at t1 and ARS score decrease (%) after correction for multiple-testing.

^b^Spearman rank test.

^c^Kruskal-Wallis test.

^d^All boys, aged 8–10 and meeting the DSM-IV ADHD criteria were eligible for participation, also if they had not officially been diagnosed with ADHD prior to the study start.

### ADHD symptoms

**Figure 2:**
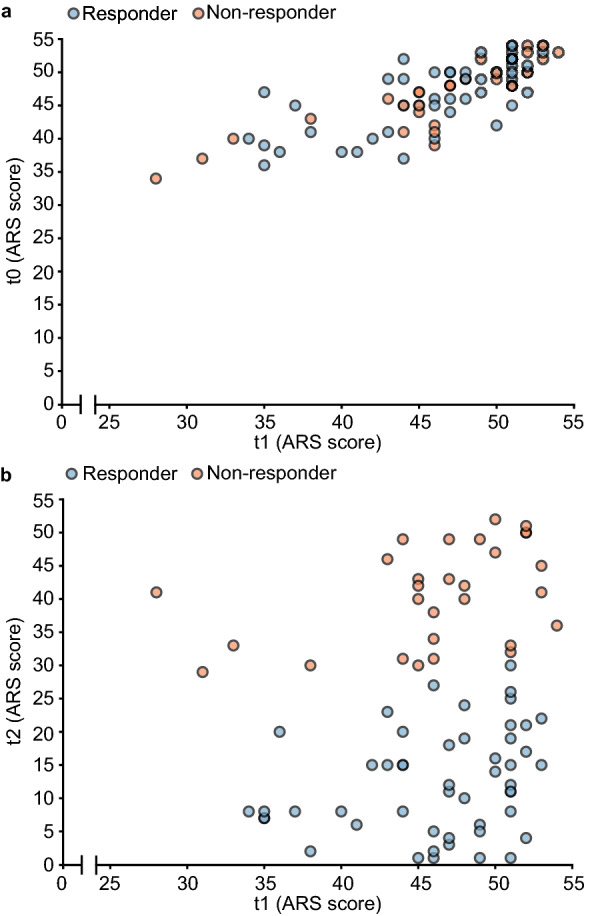
ARS scores of all participants (n = 79) at t0, t1 and t2. Blue dots represent ARS scores of responders (≥ 40% ARS score decrease at t2 compared to t1; n = 50). Red dots represent ARS scores of non-responders (< 40% ARS score decrease at t2 compared to t1; n = 29). ARS scores (minimum score = 0, maximum score = 54) at: (**a**) t0 versus t1. (**b**) t1 versus t2. See Supplementary Fig. S2 for t0 versus t1 ARS scores for all participants that started the FFD (n = 100). *ARS* Attention-deficit hyperactivity disorder (ADHD) Rating Scale, *FFD* few-foods diet, *t0* screening, *t1* before start FFD, *t2* at the end of the FFD.

ADHD symptom scores were measured using the ADHD Rating Scale (ARS). ARS scores at the screening (t0) and after the baseline period (t1, before starting the FFD) were comparable (Paired *t* test, *t* value = − 1.17, *df* = 78, n = 79, *p* = 0.25; Table 2, Fig. 2 and Supplementary Fig. S2). At t0, the mean ARS score of the 79 participants was 46.7 (SD = 5.1), very similar to the mean ARS after the baseline period (t1), which was 46.2 (SD = 5.8). At the end of the FFD (t2), the mean ARS score was 22.8 (SD = 15.6), significantly lower than the mean ARS score at t1 (Paired *t* test, *t* value = − 13.12, *df* = 78, n = 79, *p* < 0.0001). Fifty (63%) of 79 participants showed an ARS score decrease of at least 40% and were categorized as responders, with an average decrease of 73.4% (SD = 16.1%), while non-responders showed an average decrease of 10.8% (SD = 17.7%). There were no differences in ARS scores distribution between the whole group of children (n = 79) and the two subgroups included in the fMRI tasks analyses (Table 2). Unfortunately, teacher t1 and t2 data were available for only 12 children, which is too few for an analysis with sufficient statistical power.

**Table 2:** Mean ARS scores at t0, t1 and t2 for the FFD group and the subgroups included in the fMRI tasks.

	N	Mean ARS score								
		t0 (SD)	t1 (SD)	t2 (SD)	t1 versus t0			t2 versus t1		
					Cohen’s d	Difference (95% CI)	*p* value^a^	Cohen’s d	Difference (95% CI)	*p* value^a^
FFD	79	46.7 (5.1)	46.2 (5.8)	22.7 (15.6)	− 0.08	− 0.5 (− 1.3, 0.3)	0.25	− 1.99	− 23.4 (− 27.0, − 19.9)	< 0.0001
Stop-signal task	53	46.7 (4.6)	46.6 (5.4)	21.5 (15.1)	− 0.02	− 0.1 (− 1.0, 0.9)	0.87	− 2.21	− 25.1 (− 29.4, − 20.9)	< 0.0001
Flanker task	32	46.9 (4.4)	46.9 (4.9)	20.8 (16.1)	0.00	0.03 (− 1.4, 1.4)	0.96	− 2.19	− 26.2 (− 31.8, − 20.5)	< 0.0001

*ARS* attention-deficit hyperactivity disorder (ADHD) rating scale (minimum = 0, maximum = 54), *CI* confidence interval, *FFD* few-foods diet, *SD* standard deviation, *t0* screening, *t1* before start FFD, *t2* at the end of the FFD.

^a^Paired *t* test.

### Task performance

The task performance results of the stop-signal task showed that both the average reaction time (RT) for the frequent Go trials (GoRT) and the average time it took to inhibit a response for the infrequent stop-signal trials (stop-signal reaction time [SSRT]) across participants did not differ between t1 and t2 (GoRT: Paired *t* test, *t* value = 1.85, *df* = 52, n = 53, *p* = 0.07; SSRT: Paired *t* test, *t* value = − 1.73, *df* = 52, n = 53, p = 0.09; Supplementary Table S1). Furthermore, both GoRT and SSRT changes between t1 and t2 were not related to changes in ARS score (Supplementary Table S2). The Flanker task performance results showed a congruency effect, i.e., participants were slower and made more errors on incongruent than on congruent trials at both t1 and t2 (RT: Wilcoxon signed rank, z-score [t1] = 4.94, z-score [t2] = 4.34, n = 32, *p* < 0.001; error rate [ERR]: Paired *t* test, *t* value [t1] = 7.73, *t* value [t2] = 6.33, *df* = 31, n = 32, *p* < 0.001; Supplementary Table S3). At t2 the Incongruent-Congruent RT was shorter than at t1 (Wilcoxon signed rank, z-score = − 2.97, n = 32, *p* = 0.002), while the Incongruent-Congruent ERR was not different between t2 and t1 (Paired *t* test, *t* value = − 1.87, *df* = 3, n = 32, *p* = 0.07; Supplementary Table S3). The decrease in RT after versus before the FFD was not related to change in ARS score (Supplementary Table S4).

### ROI analyses

**Figure 3:**
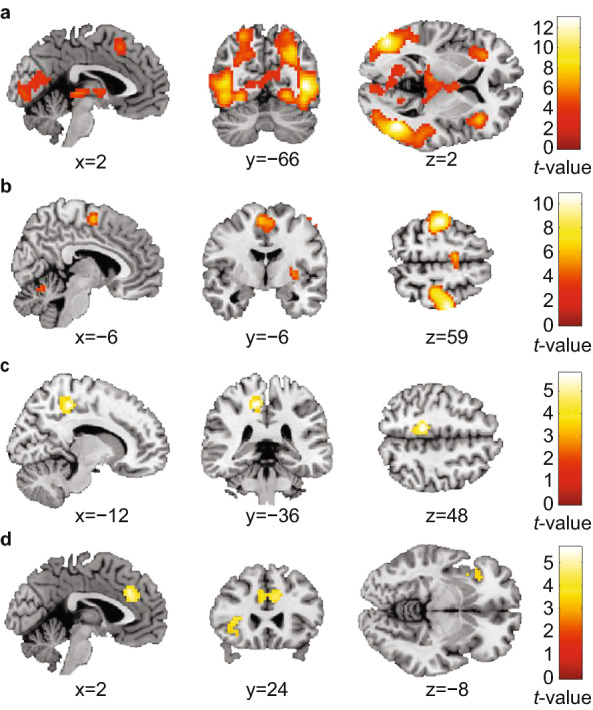
Main stop-signal task effects across subjects (n = 53) and measurements. Significant clusters at (whole-brain) pFWE < 0.05 are shown for the following contrasts: (**a**) StopSuccess > Go contrast (displayed at x = 2, y = − 66, z = 2); (**b**) Go > StopSuccess contrast (displayed at x = − 6, y = − 6, z = 59); (**c**) StopSuccess > StopFail contrast (displayed at x = − 12, y = − 36, z = 48). (**d**) StopFail > StopSuccess contrast (displayed at x = 2, y = 24, z = − 8). The cluster-defining threshold was set at *p* < 0.001 (uncorrected). The cluster extent was set at k > 55 to only show significant clusters (pFWE < 0.05, whole-brain). All mentioned coordinates are Montreal Neurological Institute (MNI) stereotaxic coordinates. The colour bars reflect peak *t* values. See Supplementary Table S5 for details per cluster. *p*_*FWE*_*-value* family-wise-error-corrected *p* value.

To investigate our primary hypothesis, functional ROIs representing the main task effect across timepoints and participants were determined, independent of the ARS response. The main task contrasts during the stop-signal task activated brain regions in frontal, parietal, temporal and occipital lobes as well as cerebellar and sub-cortical regions involved in response inhibition (Fig. 3, Supplementary Table S5). The 25 clusters activated for these main task contrasts (Supplementary Table S5) were used as primary outcome ROIs, to assess whether their mean beta weights at t2 were associated with changes in ARS scores (categorical and continuous), including previous medication use, IQ and age as covariates of non-interest. After correction for multiple comparisons, no significant effects were present (all p-values above the critical α of 0.05/25 = 0.002; Supplementary Table S6).

Analysis of the Flanker task fMRI results showed that no regions known to be related to interference inhibition and error monitoring were activated during the task. Since the Flanker task did not activate the brain as expected, no further analyses were performed.

### Whole-brain analyses

**Figure 4:**
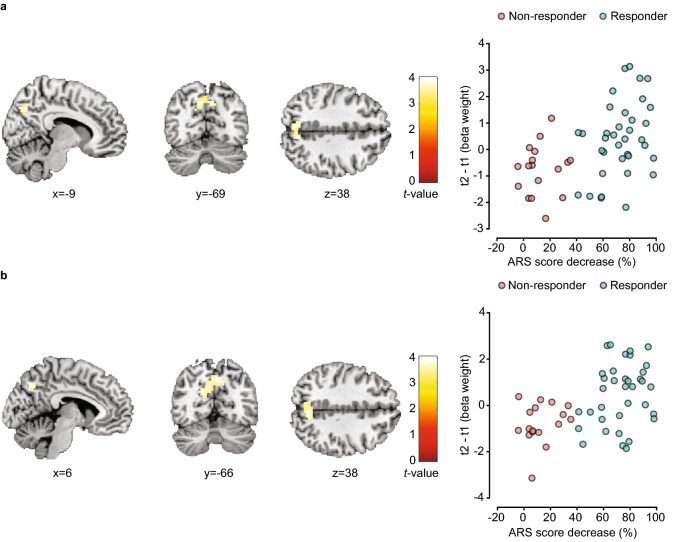
Correlation between BOLD response and the relative ARS score decrease after following an FFD (n = 53). (**a**) Significant precuneus cluster ([whole-brain] p_FWE_ = 0.015) showing a positive correlation between the absolute change (t2 − t1) in the StopSuccess > Go contrast and the ARS score decrease (100 × [t1 − t2]/t1). (**b**) Significant precuneus cluster ([whole-brain] p_FWE_ < 0.001) showing a positive correlation between the absolute change (t2 − t1) in the StopSuccess > StopFail contrast and the ARS score decrease (100 × [t1 − t2]/t1). The cluster extent was set at k > 50 to only show significant clusters (p_FWE_ < 0.05, whole-brain). All mentioned coordinates are Montreal Neurological Institute (MNI) stereotaxic coordinates. The colour bars reflect peak *t* values. See Table 3 for details per cluster. For illustration purposes, scatter plots, showing the correlation between the ARS score decrease (100 * [t1 − t2]/t1) and cluster-averaged beta weights for the StopSuccess > Go (t2 − t1) and StopSuccess > StopFail (t2 − t1) contrasts in the precuneus, were added on the right side of the corresponding brain images. Higher beta-weights correspond with a higher activation. Blue dots represent responders (≥ 40% ARS score decrease at t2 versus t1), red dots represent non-responders (< 40% ARS score decrease at t2 versus t1). *ARS* attention-deficit hyperactivity disorder (ADHD) rating scale, *FFD* few foods diet, *p*_*FWE*_*-value* family-wise-error-corrected *p* value, *t1* before start FFD, *t2* at the end of the FFD.

Our secondary aim was to investigate the changes in activation for other regions that were not expected a priori, by means of whole-brain analyses. Whole-brain fMRI analyses of the t2–t1 effects during the stop-signal task did not reveal differences in brain activation. However, when taking the changes in ARS score into account, both response inhibition contrasts showed a positive correlation between change in ARS score and change in precuneus activation, at the whole-brain, cluster-level corrected threshold p_FWE_ < 0.05 (StopSuccess > Go: general linear model [GLM], df = 50, n = 53, p_FWE_ = 0.015; StopSuccess > StopFail: GLM, *df* = 50, n = 53, p_FWE_ < 0.001; Fig. 4, Table 3).

**Table 3:** Clusters (p_FWE_ < 0.05) with their (sub-)peak coordinates showing a correlation between the absolute changes in brain activation during the stop-signal task and the relative ARS score decrease after following an FFD (n = 53).

	Hemisphere	MNI coordinates x, y, z (mm)	cluster size	peak *t*-value	p_FWE_ cluster
**StopSuccess > Go**					
Precuneus	Left	− 9, − 69, 38	67	4.18	0.015
Precuneus	Right	6, − 69, 38		4.12	
**StopSuccess > StopFail**					
Precuneus	Right	6, − 66, 38	126	4.02	< 0.001
Superior occipital gyrus	Left	− 18, − 69, 24		3.96	
Precuneus	Left	− 9, − 69, 31		3.87	

*ARS* attention-deficit hyperactivity disorder (ADHD) rating scale, *FFD* few-foods diet, *MNI coordinates* Montreal Neurological Institute stereotaxic coordinates, *p*_*FWE*_*-value* family-wise-error-corrected *p* value. Sub-peaks are indented.

### ODD symptoms

ODD was present in 58 of 79 children at t1. Fifty-seven children were included in the ODD analyses; one of the 58 participants was excluded based on an additional intervention during the FFD (parents avoided all activities that were known to incite ODD behaviour), that might have affected the ODD results at the end of the FFD. For these 57 children, the mean ODD score at t1 was 17.8. At t2, the mean ODD score was reduced to 7.6 (Paired *t* test, *t* value = − 11.4, *df* = 56, n = 57, Cohen’s d = − 1.97, difference [95% CI] = − 10.2 [− 11.9, − 8.4], *p* < 0.0001). Forty (69%) of 57 participants showed a decrease in ODD symptoms of more than 40%, with an average symptom decrease of 75.4% (SD = 17.4%). The other 17 (31%) participants showed an average symptom decrease of 9.9% (SD = 19.6%). Changes in ODD score and ARS score are strongly correlated (Spearman rank test, rho = 0.71, n = 57, *p* < 0.0001; Supplementary Fig. S3). Of the 40 ODD-responders, 32 showed symptom decreases of more than 40% in both ADHD and ODD behaviour, while eight of 40 ODD-responders were ADHD-non-responders (Supplementary Fig. S3).

### Exploratory whole brain analyses based on ODD symptoms

Thirty-nine (73.5%) of the 53 children that were included in the stop-signal task met the ODD criteria. In order to investigate whether the presence and the severity of ODD at t1 contributed to the correlation between precuneus activation and change in ARS score, we added the t1 ODD score as covariate of non-interest. The precuneus finding in the StopSuccess > Go contrast remained significant (x = 9, y = − 69, z = 38, p_FWE_[cluster] = 0.030, k = 59), however, in the StopSuccess > StopFail contrast, the precuneus finding no longer survived whole-brain correction for multiple comparisons (x = 6, y = − 66, z = 38, p_FWE_[cluster] = 0.201, k = 33, *p*[uncorr, peak] < 0.001).

## Discussion

This study is the first fMRI study investigating whether a decrease in ADHD symptoms following an FFD relates to changes in brain function in children with ADHD. The results showed no relation between brain function and behavioural changes in the ROIs representing the main task effects across subjects and time points, thereby rejecting our primary hypothesis. However, in our secondary explorative whole-brain analyses, the decrease in ADHD symptoms after following an FFD was significantly correlated with an increase in inhibition-related activation of the precuneus during a stop-signal task, pointing at an underlying neurocognitive mechanism.

Fifty (63%) of 79 participants were responders, with an average ARS score decrease of 73%. This response percentage is commensurate with the response observed in previous studies applying an FFD, assessed by different research groups^10,11,22,23^ and raters^24^. An FFD is a short-duration nutritional intervention to assess whether food is a trigger for ADHD symptoms. Commonly, FFD responders then proceed to a challenge phase to identify foods that trigger ADHD, eventually resulting in a personalized diet excluding only those foods that the child reacts to^9^. Long-term studies applying a few-foods approach, followed by double-blind challenges, have shown that a relapse in behaviour may occur after eating any specific type of food, whether it be major allergens, minor allergens, or even everyday foods like beef, chicken, corn, potatoes and cucumber^22,23^. Also, children often reacted to more than one food^22,23^. This knowledge underlines the importance of applying an FFD as a standardized intervention in further research into the effect of food on ADHD.

The fMRI results of the current study, i.e., an increase in precuneus activation in FFD responders, are in line with results from medication studies that demonstrated that methylphenidate increased precuneus activity in boys with ADHD during the stop-signal task or a sustained attention task^16,25^. Moreover, the present results are also in agreement with other fMRI studies in ADHD that reported reduced activation of the precuneus in children with ADHD *versus* matched controls during attention and stop-signal tasks^15^. Although the precuneus is not a typical region involved in response inhibition, it is a region involved in visuospatial processes^26^ that contribute more generally to performing a visual task such as our stop-signal task. However, an increased activation of the precuneus in ADHD compared to matched controls during a vigilance task has also been reported^27^, suggesting that the precuneus may be under- or over-activated in ADHD depending on the type of task that is used. The precuneus is involved in visuospatial attention, but is also part of the brain’s default mode network; a network of brain areas that is active when an individual is awake and alert but not actively engaged in a task^26^. Functional MRI resting state studies investigating the default mode network showed an association between decreased functional connectivity and ADHD^28^. However, our study did not investigate the activation of the precuneus under resting state conditions, which should be addressed in future research.

Contrary to the observation that brain function was correlated with behavioural improvement following an FFD, no relationships were detected between task performance and changes in ARS score. This result is in agreement with studies investigating the effect of medication on ADHD, often detecting no differences in task performance between the medication and placebo group^29^. Indeed, changes in brain activation have been considered to be better predictors for ADHD treatment response than differences in task performance^30,31^.

Forty of 57 children included for ODD analysis were ODD-responders, 32 of these responders were also ADHD-responder, i.e., showing ADHD and ODD symptom decreases of at least 40%. These results indicate that nutritional intervention may not only have beneficial effects on ADHD, but also on ODD, which is in line with previous research results^10^. Exploratory post-hoc fMRI analyses did not provide indications that the increase of precuneus activation in children showing a decrease of ADHD symptoms was driven by co-morbid ODD. Indeed, post-hoc analyses showed that we could consistently reproduce our ARS precuneus finding independently of comorbid ODD. This is an important observation, showing that our results are uniformly applicable to children with ADHD, whether or not suffering from comorbid ODD.

In the ROIs that were based on the main task effects across subjects and time points, no significant associations of brain function and behavioural changes were found, even though previous medication studies in ADHD have found effects in fronto-parietal regions more typically involved in executive control^16,25^. We cannot explain well why task-related regions did not show effects in our study. However, an ROI analysis does limit the outcomes to specific brain regions, while the observed effects can also be found in regions that are not activated by the task across subjects and time points. For instance, a dietary intervention could affect brain function by compensatory changes in brain regions that are not generally involved in a task. Moreover, group task effects—that our ROIs were based upon—can miss out on important inter-individual variability^32,33^. Our whole-brain analysis, on a cluster-level corrected threshold, revealed that the decrease of ADHD symptoms was significantly correlated with increased precuneus activation in two different stop-signal contrasts.

A limitation of the current study was the lack of a control group and blinded design. An expectancy or a disappointment effect, both on parental ratings and the child’s behaviour, may have affected the behavioural outcomes. However, our purpose was not to evaluate whether or not the FFD affects the behaviour of a child with ADHD, but to investigate whether the observed behavioural improvements after following an FFD are correlated with changes in brain activation, comparably to the study goal of Stevens et al. (2015)^34^. Therefore, we were able to apply the most restricted diet, i.e., the FFD, in order to achieve the best possible behavioural response^6^. Another limitation of the present study was that the study population was confined to boys of a narrow age range, which hampers generalization of the results to girls and other age groups. Furthermore, 26 (33%) of the participants could not be included in the stop-signal task analysis, due to movement in the scanner or technical issues. However, both the ARS scores and the baseline characteristics were not different between the complete study population (n = 79) and the children included in the stop-signal task analysis (n = 53). Finally, although our study clearly identified activation of the precuneus to be related to ADHD symptom improvement after following an FFD, the underlying molecular mechanism that explains this activation remains to be deciphered.

This study showed that the parent-reported behavioural improvements in children with ADHD following an FFD, were reflected in the brain activity of the children. The correlation between the increase in inhibition-related activation of the precuneus and the clinical symptom decrease at the end of the FFD, objectively points to an underlying neurocognitive mechanism. Future research should not only focus on further investigating the role of the precuneus in food-induced ADHD, but also on the underlying biological mechanism and the impact of the gut-brain axis, which is highly modifiable by diet^4^. Our study results are clinically relevant for children with ADHD and for the scientific community, providing new leads towards improved understanding of the impact of nutrition on ADHD.

## Subjects and methods

### Participants

Participants in the Biomarker Research in ADHD: the Impact of Nutrition (BRAIN) study were recruited via the media and healthcare institutions. Eligibility for participation in the study was assessed during a personal intake interview with the parents. Inclusion criteria were right-handed boys, aged ≥ 8 and ≤ 10 years, and meeting the Diagnostic and Statistical Manual of Mental Disorders, fourth edition (DSM-IV) criteria of ADHD. Exclusion criteria were (1) diagnosis of autism spectrum disorder, developmental coordination disorder, chronic gastrointestinal disorder, autoimmune disorder, dyslexia or dyscalculia, (2) premature birth (< 36 weeks) and/or known oxygen deprivation during birth, (3) vegetarian/vegan, (4) IQ < 85, (5) use of systemic antibiotics, antifungals, antivirals or antiparasitics in the past 6 months, (6) insufficient command of the Dutch language by either parents or child, (7) family circumstances that may compromise following or completion of the diet, and (8) having a contraindication to MRI scanning. After t1, participants were withdrawn or excluded from the study if the child or family did not comply with the instructions, or if family circumstances interfered with study compliance. Participants and/or the parents of the participants could withdraw from the study at any time. After the procedures had been fully explained, all parents gave written informed consent, and all children gave written assent. Based on the sample size estimates per outcome variable of the BRAIN study (i.e., neural activation, phenylalanine and tyrosine plasma levels and gene abundance in stool microbiota), as calculated in the published protocol of this study^21^, 100 children were included. The required samples size for the main hypothesis of this manuscript was estimated at 46^21^.

### Study design

Children meeting the BRAIN study criteria attended a screening session at Wageningen University and Research, The Netherlands, for further assessments (t0). The ADHD diagnosis was established by a trained paediatrician. An abbreviated IQ test based on the Wechsler Intelligence Scale for Children-III was conducted if there were no IQ test results available from the past year, and family circumstances and baseline ADHD symptom scores were assessed. Children were familiarized with the scanning procedure in a mock scanner.

Children meeting the criteria for participation started with a 2-week baseline period, during which they stayed on their regular diet, while parents kept a diary meticulously documenting their child’s food, behaviour and activities. The period between t0 and t1 was called the baseline period. At t1, after the baseline period, the first fMRI scan was performed, ADHD symptom scores were assessed again, and parents received an elaborate explanation of the FFD^21^. Then, following a 1-week transition period applying a gradually adapted diet to habituate to a different eating pattern, the children started the FFD, which lasted 32 or 33 days (starting on a Monday and ending on a Thursday or Friday, almost 5 weeks later). The most stringent FFD consisted of rice, turkey, vegetables (cabbage [white, green, Chinese, red], beet, cauliflower, borecole, swede, sprouts, lettuce), pears, olive oil, ghee, salt, rice drink with added calcium and water. During the first 2 weeks of the diet, the FFD was extended with some other foods, allowing lamb, butter and small portions of wheat, corn, potatoes, some fruits, and honey^10,24^. If no behavioural improvement was reported within 2 weeks, the extended FFD was gradually adapted to the most stringent FFD^22,23^. At the end of the FFD both fMRI and ADHD symptom score assessments were repeated (t2).

### Behavioural scores

ADHD symptom scores before and after the FFD were measured using the ARS, consisting of 18 ADHD symptoms and applying a four-point scale (0 = at the most twice a week, 1 = several times a week, 2 = once a day, and 3 = several times a day; maximum score = 54)^35,36^. This questionnaire was completed by the parents at t0, t1 and t2; if possible, teachers completed the ARS at t1 and t2. Prior to the measurements, parents and teachers were asked to focus on the child’s behaviour during the past week only. The percentage change in ARS score at t2 relative to t1 (100 × [t1 − t2]/t1) was used to assess a child's response to the FFD. Children with an ARS reduction of at least 40% at t2 compared to t1 were designated responders^21^. ODD, a co-morbid disorder commonly occurring in children with ADHD, was assessed at t1 and t2 by means of a structured psychiatric interview using the eight ODD-symptoms (DSM-IV), applying a four-point scale (0 = less than once a week, 1 = once or twice a week, 2 = three times a week, and 3 = more than three times a week; maximum score = 24). ODD criteria were met when at least four out of eight symptoms occurred at least three times a week. Children with an ODD symptom score reduction of at least 40% at t2 compared to t1, were designated ODD responders (22).

### MRI acquisition

The fMRI tasks were programmed in Presentation (www.neurobs.com). An optical button box with four buttons was used. Participants were instructed to only use the left and right button, pressing the left button with the left hand and the right button with the right hand. A screen was used in the scanner to present the tasks to the participants. Before the scans, participants were familiarized with the tasks. On both fMRI-assessment days (t1 and t2), participants were scanned, at the same time of day, on a 3 T-Siemens Magnetom Verio with a 32-channel head coil. Participants underwent: (1) a *functional MRI* scan during a stop-signal task (duration ± 8 min), (2) an *anatomical MRI* scan (duration ± 5 min), and (3) a *functional MRI* scan during a Flanker task (duration ± 5 min). See Supplementary methods for the fMRI scan parameters.

### Task performance

The stop-signal task^37^ was used to assess response inhibition, and the Flanker task^38^ was used to assess interference inhibition and associated error monitoring (Supplementary Fig. S1, Supplementary methods). Performance during the stop-signal task was assessed with the GoRT, the SSRT, and the GoRT variability (GoRTvar = SD[GoRT]/mean[GoRT]). The interval between the Go signal and the Stop signal in the Stop trials, i.e., the stop-signal delay, became shorter or longer in steps of 50 ms depending on each participant’s performance, i.e., after a successful or unsuccessful inhibition respectively. We calculated the SSRT by subtracting the average stop-signal delay at which participants achieved 50% of inhibition from the average GoRT^39^. Performance during the Flanker task was measured with the RT, RT variability (RTvar = SD[RT]/mean[RT]) and ERR of incongruent and congruent trials. The main congruency effect of the Flanker task was assessed with the contrast of incongruent *versus* congruent trials.

### MRI pre-processing

Anatomical and functional MRI pre-processing was done using fmriprep^40^, as described in more detail in the Supplementary methods. In brief, pre-processing steps included co-registration of the functional to the structural (anatomical) image, motion correction (realignment), slice time correction, spatial normalization and spatial smoothing. Time-series of components that were identified as noise by independent component analysis (ICA-AROMA)^41^ were collected as potential noise regressors for the first level fMRI analysis (see next section).

### Functional MRI analysis

At the first, subject-specific level, we specified a model with regressors of interest (task-related events) and regressors of non-interest (noise regressors). For the stop-signal task, this model included three event regressors of interest per session (t1 and t2), reflecting: 1. Go trials; 2. StopSuccess trials, 3. StopFail trials. Missed Go trials were added as regressor of non-interest. Other noise regressors included the framewise displacement^42^, the six rigid-body realignment parameters, the mean signal from the cerebrospinal fluid, and four physiological regressors (CompCor)^43^. Finally, we added the time courses of the independent noise components of ICA-AROMA as regressors of non-interest, with the restriction that we only included those that could not regress out significant amounts of task-related (thus, relevant) variance. All task regressors were convolved with the canonical hemodynamic response function and high pass filtered (128 s). At the first level, two contrasts were specified to assess response inhibition during the stop-signal task: StopSuccess > StopFail and StopSuccess > Go. To assess performance monitoring and error processing, we use the contrast: StopFail > StopSuccess contrast. To generate a motor contrast, we used: Go > StopSuccess.

The Flanker task was analysed in the same way as the stop-signal task, except that different regressors of interest were used: 1. congruent trials; 2. Incongruent trials. Response conflict during the Flanker task was assessed using the contrast: Incongruent > Congruent.

For a full description of the first level analysis, see Supplemental methods. The first level contrast images were subsequently used in a second level (group) random effects analysis to assess task effects across participants and sessions, as well as the effects of FFD response.

The primary objective of the BRAIN study was to explore the relation between ARS score changes and BOLD response changes after the FFD in ROIs during task performance. To this end, functional ROIs were used, defined by the main task effect, i.e., across participants and timepoints, independent of the ARS score decrease (%). The functional ROIs were determined using a GLM for each of four stop contrasts (StopSuccess > / < StopFail and StopSuccess > / < Go) and two Flanker contrasts (Congruent > / < Incongruent) across sessions (i.e., t1 and t2) and across participants. ROIs were defined as the clusters showing a main task effect using a family-wise-error-corrected p-value (p_FWE_) < 0.05 (cluster level). Regionally averaged beta weights were extracted for both timepoints from all voxels in the identified ROIs using MarsBar^44^.

Our secondary objective was to explore the relation between ARS score changes and BOLD response after the FFD over the whole brain. In this exploratory whole-brain analysis, a GLM was used for each of the six above-mentioned contrasts, with the percentage change in ARS score (100 * [t1 − t2]/t1) as covariate of interest in the voxel-wise t2 − t1 difference. Both positive and negative between-subject correlations were investigated. To explore the effects of ODD symptom severity at t1 on the outcomes of the above mentioned whole-brain analyses, we repeated the analyses with the t1 ODD score per subject as a covariate of non-interest.

To correct for the possibility that ADHD symptom severity is related to movement during the scan, potentially causing a noise bias, the average movement per subject (i.e., mean framewise displacement) across t1 and t2, was added in all group-level analyses as a covariate of non-interest. The results of these group-level fMRI analyses were assessed at the cluster level, by applying p_FWE_ < 0.05 as threshold for multiple comparisons over the whole brain (cluster-defining threshold at *p* < 0.001, uncorrected). All analyses were performed using the SPM software package (SPM12).

### Statistical analysis

All statistical analyses described in this section were carried out using SAS® (version 9.4).

ARS scores at t0 and t1 were compared using a paired t-test. The same test was used to compare ARS scores before (t1) and after (t2) the FFD intervention. The ODD scores at t1 and t2 were compared by a paired *t* test including only those children meeting the ODD criteria at t1. If the assumption of normality was violated then non-parametric tests were applied (paired Wilcoxon signed rank test or Kruskal–Wallis test). Normality was assessed by the Shapiro–Wilk test.

The task performance results obtained at t2 and t1 were compared with a paired *t* test if the differences (results at t2 minus results at t1) were normally distributed, if not, a Wilcoxon signed rank test was used. The association between task performance results at t2 and the percentage change in ARS score at t2 compared to t1 (on both a categorical and continuous scale) was assessed using a general linear model (ANCOVA), including the task performance results at t1 as covariate of non-interest. If the model residuals were not normally distributed, a nonparametric Kruskal–Wallis test was applied (ARS score change on a categorical scale) or a Spearman rank correlation (ARS score change on a continuous scale) was estimated.

The t2 beta weights extracted from the ROIs in the fMRI analysis were further analysed using a general linear model (ANCOVA) with the change in ARS score (categorical or continuous) as covariate of interest and including t1 beta weights, age, IQ, and previous use of ADHD medication as covariates of non-interest.

### Study registration, approval and ethics statement

This study is registered as “Biomarker Research in ADHD: the Impact of Nutrition (BRAIN)” at ClinicalTrials.gov, number NCT03440346 (22/02/2018). It was approved by the Medical Research and Ethics Committee of Wageningen University (NL63851.081.17, application 17/24); the protocol has been published^21^ and pre-registered at Openscience (https://osf.io/6aeq3). The authors assert that all procedures contributing to this work comply with the ethical standards of the relevant national and institutional committees on human experimentation and with the Helsinki Declaration of 1975, as revised in 2008.

### Blinding

In this open-label intervention study all participants received the FFD, and parents, children and researchers involved in applying the FFD were not blinded. However, during the data quality checks, pre-processing and subject-level fMRI analysis, data were evaluated by researchers blinded to the ADHD symptom scores on both t1 and t2 (i.e., they were blinded regarding respondership status).

### Role of the funding source

The sponsor, Porticus, was not involved in study design and execution, data analysis and its interpretation, writing the manuscript and the submission for publication.

## Supplementary Information

Supplementary Information.

## Data Availability

Source MRI data was converted to the Brain Imaging Data Standard (BIDS)^45^ and converted using the open source BIDScoin (https://github.com/Donders-Institute/bidscoin) toolbox. Data resulting from this study can be downloaded from the Data Archiving and Networked Services (DANS) of the Royal Netherlands Academy of Arts and Sciences and the Netherlands Organization for Scientific Research (https://doi.org/10.17026/dans-xzf-wh36).
