# Supplementary material for: Correlation between brain function and ADHD symptom changes in children with ADHD following a few-foods diet: an open-label intervention trial
Source: Sci Rep. 2021 Nov 12;11:22205. doi: 10.1038/s41598-021-01684-7 (PMC8589974; doi:10.1038/s41598-021-01684-7)
Supplement: Supplementary file 1 — Supplementary Information. [file 41598_2021_1684_MOESM1_ESM.pdf]

Supplementary Material

Correlation between changes in brain function and ADHD symptoms in children with ADHD following a few-foods diet: an open-label intervention trial

Saartje Hontelez, Tim Stobernack, Lidy M. Pelsser, Peter van Baarlen, Klaas Frankena, Martine M. Groefsema, Michiel Kleerebezem, Rob Rodrigues Pereira, Elbrich M. Postma, Paul A. M. Smeets, Marion A. Stopyra, Marcel P. Zwiers, Esther Aarts

Table of Contents

Supplementary Methods 3

MRI scans 3

Functional MRI task description 3

Anatomical MRI data pre-processing 3

Functional MRI data pre-processing 4

First level fMRI analysis 4

Contrasts applied in second level fMRI analysis 5

References 5

Supplementary Tables 7

Supplementary Figures 14

Supplementary Methods

MRI scans

The following scan sequences were used: *T_2*-weighted functional MRI scans* for the stop-signal and the Flanker task; a 2D Echo-Planar Imaging sequence, with GRAPPA factor 2, repetition time [TR] = 2030 ms, echo time [TE] = 30 ms, 75° flip angle, field of view [FOV] = 192 x 192 mm, 37 axial 3-mm slices with 17% gap, acquired in interleaved descending order. Three hundred and fifty volumes were acquired for the stop-signal task and 293 for the Flanker task. A *T_1-weighted anatomical scan;* an MP-RAGE sequence, with TR = 1900 ms, TE = 2.26 ms, 9° flip angle, FOV = 256 × 256 mm, 192 sagittal slices, voxel size = 1 × 1 × 1 mm), alternatively, a 'fast' sequence with TR = 5.8 ms, TE = 2.06 ms, 11° flip angle, FOV = 256 x 256 mm, 176 sagittal slices, voxel size = 1 × 1 × 1 mm.

Functional MRI task description

Prior to the stop-signal task and the Flanker task, the children were shown an explanation of the task and performed a set of test trials. During the stop-signal task, 80% of the trials were Go trials, requiring a left or a right response depending on the direction of the horizontal arrow, and 20% of the trials were Stop trials, with a vertical arrow following a left or right pointing arrow quickly in time, and requiring an inhibition of the response (supplementary figure 1). The interval between horizontal (Go-signal) and vertical arrows (Stop-signal) in the Stop trials became shorter or longer in steps of 50 ms depending on each participant's performance (a.k.a. a staircase procedure), ensuring 50% of correctly inhibited (StopSuccess) and 50% failed stop (StopFail) trials for each participant. The interval between trials was jittered between 1.6 s and 2.0 s, with an average of 1.8 s. Arrows were on the screen for 500 ms. Forty Stop trials (20 of them appearing after a left pointing arrow and 20 appearing after a right pointing arrow) were pseudo-randomly intermixed with 156 Go trials (78 left pointing arrows and 78 right pointing arrows), and at least three repetition times (TRs) apart from each other.

For each of the Flanker task trials, five flanking arrows were shown, and a left or right response was required, depending on the direction of the central arrow (supplementary figure 1). Half of the trials were congruent trials with five arrows pointing in the same direction, and half of the trials were incongruent trials with the four flanking arrows pointing in the opposite direction. The interval between trials was jittered between 2.0 s and 6.0 s, with an average of 4.0 s. Arrows were on the screen for 750 ms. Thirty incongruent trials (15 of them a left response and 15 of them requiring a right response) were pseudo-randomly intermixed with 30 congruent trials (15 requiring right responses and 15 requiring left responses).

Anatomical MRI data pre-processing

All T_1-weighted (T1w) images were corrected for intensity non-uniformity (INU) with N4BiasFieldCorrection [1], distributed with ANTs 2.2.0 [2]. The T1w-reference was then skull-stripped with a Nipype implementation of the antsBrainExtraction.sh workflow (from ANTs), using OASIS30ANTs as target template. Brain tissue segmentation of cerebrospinal fluid (CSF), white-matter (WM) and gray-matter (GM) was performed on the brain-extracted T1w using FAST (FSL 5.0.9) [3]. A T1w-reference map was computed after registration of all T1w images (after INU-correction) using mri_robust_template (FreeSurfer 6.0.1) [4]. Brain surfaces were reconstructed using recon-all (FreeSurfer 6.0.1) [5], and the brain mask estimated previously was refined with a custom variation of the

method to reconcile ANTs-derived and FreeSurfer-derived segmentations of the cortical gray-matter of Mindboggle [6]. Volume-based spatial normalization to standard space (MNI152NLin2009cAsym) was performed through nonlinear registration with antsRegistration, using brain-extracted versions of both T1w reference and the T1w template.

Functional MRI data pre-processing

For all fMRI runs of each session, a reference volume and its skull-stripped version were first generated using an adapted version of the fMRIPrep pipeline [7]. The BOLD reference was then co-registered to the T1w reference using bbregister (FreeSurfer) which implements boundary-based registration [8]. Co-registration was configured with nine degrees of freedom to account for distortions remaining in the BOLD reference. Head-motion parameters with respect to the BOLD reference (transformation matrices, and six corresponding rotation and translation parameters) were estimated before any spatiotemporal filtering in mcflirt (FSL 5.0.9) [9]. BOLD runs were slice-time corrected using 3dTshift from AFNI 20160207 [10]. The BOLD time-series were resampled to surfaces on the fsaverage5 space. The BOLD time-series (including slice-timing correction when applied) were resampled onto their original, native space by applying a single, composite transform to correct for head-motion and susceptibility distortions. These BOLD time-series were then resampled into the standard space of the T1w image, generating the spatially-normalized, pre-processed BOLD runs.

Automatic removal of motion artefacts using independent component analysis (ICA-AROMA) [11] was performed on the pre-processed BOLD on MNI space time-series after removal of non-steady state volumes and spatial smoothing with an isotropic, Gaussian kernel of 6mm FWHM (full-width half-maximum). Time-series of components that were identified as noise were collected as potential noise regressors for the 1st-level fMRI analysis (see further below). Additional confounding time-series were calculated based on the pre-processed BOLD, i.e. the framewise displacement (FD; Nipype implementation following Power *et al.* 2014) [12], the six rigid-body realignment parameters and the mean signal from the cerebrospinal fluid. Lastly, a set of physiological regressors were extracted to allow for principal component-based noise correction (CompCor) [13]. Components were estimated after high-pass filtering the pre-processed BOLD time-series (using a discrete cosine filter with 128 s cut-off) for the anatomical CompCor variant (aCompCor): the top 5% variable voxels within a non-GM subcortical mask that is obtained by heavily eroding a CSF and WM mask. For each CompCor decomposition, the five components with the largest singular values were included as noise regressors in the fMRI 1st-level analysis.

First level fMRI analysis

At the first level, individual, subject-specific data of both experimental sessions (t1 and t2) were analysed using a single fixed effects model. For the stop-signal task, this model included three event regressors of interest per session (thus, six regressors of interest in total), reflecting: 1. Go trials; 2. StopSuccess trials, 3. StopFail trials. Onsets of the Go regressor events reflected the moment the Go pictures (left or right pointing arrow) were presented on the screen, of trials in which the participant gave a button response and in which no stop-signal appeared. The onsets of the StopSuccess and StopFail regressors were reflecting the moment the Stop pictures (upwards pointing arrow) were presented on the screen, of trials in which participants successfully inhibited their response (StopSuccess) or trials in which participants failed to inhibit their response (StopFail). All event durations were

put to zero (i.e. stick functions). A regressor of non-interest was added (GoMiss), reflecting onsets of the Go trials for which the participant had given no button box response. We also added a regressor of non-interest reflecting signal variation in cerebrospinal fluid regions. Moreover, as regressors of non-interest, we added six rigid-body transformation parameters (three translations [x, y, z] and three rotations [pitch, roll, jaw], as well as the framewise displacement (FD; the sum of the absolute values of the derivatives of the six realignment parameters) and four anatomical noise regressors (CompCor) [13]. Finally, we added the time courses of the independent noise components of ICA-AROMA as regressors of non-interest, with the restriction that we only included those that could not regress out significant amounts of task-related (thus, relevant) variance. To achieve this, we used the ICA components identified as motion by ICA-AROMA in a multiple regression analysis with the task regressors as predictors and the component time courses as dependent variables. In case the fitted R^2 of a component was higher than 5%, they were not included in the first level model as noise regressors (i.e. regressor of non-interest). Note that therefore the number of these ICA-AROMA regressors varied per participant and session. All task regressors were convolved with the canonical hemodynamic response function. High pass filtering (128 s) was applied to the time series of the functional images to remove low-frequency drifts and correction for serial correlations was done using an autoregressive AR(1) model. First level parameter estimates for the regressors of interest, derived from the mean least-squares fit of the model to the data, were used to generate the first level Stop contrast images.

The Flanker task was analysed in the same way as the stop-signal task, except that different regressors of interest were used: 1. congruent trials; 2. Incongruent trials.

Contrasts applied in second level fMRI analysis

The first level contrast images were subsequently used in a second level (group) random effects analysis to assess task effects across participants and sessions, as well as the effects of FFD response. Response inhibition during the stop-signal task can be assessed using two different contrasts: StopSuccess>StopFail and StopSuccess>Go. The contrast StopSuccess>StopFail provides optimal control for stimulus-driven processing (i.e. presentation of the stop-signal). The contrast StopSuccess>Go provides optimal control for the outcome of the trial (i.e. both successful). To assess performance monitoring and error processing, we also looked at the StopFail>StopSuccess contrast. Go>StopSuccess provides a motor contrast (i.e. button presses versus no button presses). The response conflict of the Flanker task was assessed using the Incongruent>Congruent contrast.

References

1 Tustison, N. J. *et al.* N4ITK: improved N3 bias correction. *IEEE Trans Med Imaging* **29**, 1310-1320 (2010).

2 Avants, B. *et al.* Multivariate analysis of structural and diffusion imaging in traumatic brain injury. *Acad Radiol* **15**, 1360-1375 (2008).

3 Zhang, Y., Brady, M. & Smith, S. Segmentation of brain MR images through a hidden Markov random field model and the expectation-maximization algorithm. *IEEE Trans Med Imaging* **20**, 45-57 (2001).

4 Reuter, M., Rosas, H. D. & Fischl, B. Highly accurate inverse consistent registration: a robust approach. *NeuroImage* **53**, 1181-1196 (2010).

5 Dale, A. M., Fischl, B. & Sereno, M. I. Cortical surface-based analysis. I. Segmentation and surface reconstruction. *NeuroImage* **9**, 179-194 (1999).

6 Klein, A. *et al.* Mindboggling morphometry of human brains. *PLoS Comput Biol* **13**, e1005350, 10.1371/journal.pcbi.1005350 (2017).

7 Esteban, O. *et al.* fMRIPrep: a robust preprocessing pipeline for functional MRI. *Nat Methods* **16**, 111-116 (2019).

8 Greve, D. N. & Fischl, B. Accurate and robust brain image alignment using boundary-based registration. *NeuroImage* **48**, 63-72 (2009).

9 Jenkinson, M., Bannister, P., Brady, M. & Smith, S. Improved optimization for the robust and accurate linear registration and motion correction of brain images. *NeuroImage* **17**, 825-841 (2002).

10 Cox, R. W. & Hyde, J. S. Software tools for analysis and visualization of fMRI data. *NMR Biomed* **10**, 171-178 (1997).

11 Pruim, R. H. R. *et al.* ICA-AROMA: A robust ICA-based strategy for removing motion artifacts from fMRI data. *NeuroImage* **112**, 267-277 (2015).

12 Power, J. D. *et al.* Methods to detect, characterize, and remove motion artifact in resting state fMRI. *NeuroImage* **84**, 320-341 (2014).

13 Behzadi, Y., Restom, K., Liau, J. & Liu, T. T. A component based noise correction method (CompCor) for BOLD and perfusion based fMRI. *NeuroImage* **37**, 90-101 (2007).

Supplementary Tables

Supplementary Table 1 Comparison of mean reaction times during stop-signal task performance (n=53) at t2 *versus* t1

	**t1 (SD)**	**t2 (SD)**	**p-value[1] (t1 *versus* t2)**
GoRT	0.67 (0.14)	0.69 (0.16)	0.07
SSRT	0.41 (0.10)	0.39 (0.10)	0.09

FFD=few-foods diet. GoRT=go reaction time. RT=reaction time. SD=standard deviation. SSRT=stop-signal reaction time. t1=before start FFD. t2=at the end of the FFD.

[1]Paired t-test.

Supplementary table 2 Comparison of mean reaction times during stop-signal task performance (n=53) at t2 *versus* t1, in relation to the relative ARS score decrease[1]

	ARS score decrease (categorical)					ARS score decrease (continuous)		
	Responder[2] n=36		Non-responder[3] n=17					
	t1 (SD)	t2 (SD)	t1 (SD)	t2 (SD)	p-value[4]	t1 (SD)	t2 (SD)	p-value[5]
GoRT	0.67 (0.15)	0.69 (0.17)	0.67 (0.12)	0.68 (0.14)	0.36	0.67 (0.14)	0.69 (0.16)	0.18
GoRTvar	0.30 (0.07)	0.28 (0.06)	0.30 (0.05)	0.29 (0.05)	0.54	0.30 (0.07)	0.28 (0.05)	0.52
SSRT	0.41 (0.11)	0.40 (0.11)	0.41 (0.10)	0.38 (0.09)	0.41	0.41 (0.10)	0.39 (0.10)	0.60

ARS=Attention-deficit hyperactivity disorder (ADHD) Rating Scale. FFD=few-foods diet. GoRT=go reaction time. GoRTvar=SD(GoRT)/mean(GoRT). SD=standard deviation. SSRT=stop signal reaction time. t1=before start FFD. t2=at the end of the FFD.

[1]ARS score decrease=100x(t1-t2)/t1 ARS score.

[2]Responders show ≥40% ARS score decrease at t2 compared to t1.

[3]Non-responders show <40% ARS score decrease at t2 compared to t1.

[4]GLM: t2=t1+response status (categorical).

[5]GLM: t2=t1+ARS score decrease in percentage (continuous).

Supplementary table 3 Comparison of mean reaction times during Flanker task performance (n=32) at t2 *versus* t1

	**Reaction time**			**Error rate**		
	**t1 (SD)**	**t2 (SD)**	**p-value (t1 *versus* t2)**	**t1 (SD)**	**t2 (SD)**	**p-value (t1 *versus* t2)**
Congruent	702.0 (107.7)	729.7 (122.9)	0.08[1]	0.025 (0.024)	0.024 (0.034)	0.57[2]
Incongruent	851.7 (224.2)	820.4 (157.6)	0.20[2]	0.184 (0.121)	0.149 (0.116)	0.054[1]
Incongruent - Congruent	149.8 (167.1)	90.7 (98.1)	0.002[2]	0.159 (0.118)	0.125 (0.112)	0.07[1]
p-value (congruent *versus* incongruent)	<0.001[b]	<0.001[b]		<0.001[a]	<0.001[a]	

FFD=few-foods diet. SD=standard deviation. t1=before start FFD. t2=at the end of the FFD.

[1]Paired t-test.

[2]Wilcoxon signed rank (t1 *versus* t2, paired).

Supplementary table 4 Comparison of mean reaction times during Flanker task performance (n=32) at t2 *versus* t1, in relation to the relative ARS score decrease[1]

	ARS score decrease (categorical)					ARS score decrease (continuous)		
	Responder[2], n=22		Non-responder[3], n=10		p-value	t1 (SD)	t2 (SD)	p-value
	t1 (SD)	t2 (SD)	t1 (SD)	t2 (SD)				
**RT**								
Congruent	692.6 (98.5)	732.4 (116.6)	722.4 (129.0)	723.8 (142.2)	0.33[4]	702.0 (107.7)	729.7 (122.9)	0.97[4]
Incongruent	830.3 (195.7)	805.3 (121.5)	898.9 (283.1)	853.6 (222.0)	0.57[5]	851.7 (224.2)	820.4 (157.6)	0.94[6]
Incongruent - Congruent	137.6 (166.5)	72.9 (81.1)	176.5 (174.1)	129.9 (123.8)	0.11[4]	149.8 (167.1)	90.7 (98.1)	0.68[4]
**RTvar**								
Congruent	0.234 (0.077)	0.290 (0.190)	0.190 (0.056)	0.226 (0.066)	0.63[5]	0.220 (0.073)	0.269 (0.163)	0.06[6]
Incongruent	0.242 (0.088)	0.209 (0.075)	0.223 (0.085)	0.251 (0.114)	0.10[5]	0.236 (0.086)	0.222 (0.090)	0.18[6]
Incongruent - Congruent	1.135 (1.777)	8.280 (31.758)	0.478 (0.340)	0.972 (1.288)	0.68[5]	0.930 (1.506)	5.996 (2.373)	0.55[6]
**ERR**								
Congruent	0.026 (0.025)	0.026 (0.034)	0.023 (0.022)	0.020 (0.036)	0.98[5]	0.025 (0.024)	0.024 (0.034)	0.94[6]
Incongruent	0.183 (0.122)	0.150 (0.114)	0.187 (0.124)	0.147 (0.127)	0.88[4]	0.184 (0.120)	0.149 (0.116)	0.91[4]
Incongruent - Congruent	0.158 (0.121)	0.124 (0.112)	0.163 (0.117)	0.127 (0.116)	0.98[4]	0.159 (0.118)	0.125 (0.112)	0.98[4]

ARS=Attention-deficit hyperactivity disorder (ADHD) Rating Scale. ERR=error rate. FFD=few-foods diet. RT=reaction time. RTvar=SD(RT)/mean(RT). SD=standard deviation. t1=before start FFD. t2=at the end of the FFD.

[1]ARS score decrease=100x(t1-t2)/t1 ARS score.

[2]Responders show ≥40% ARS score decrease at t2 compared to t1.

[3]Non-responders show <40% ARS score decrease at t2 compared to t1.

[4]GLM: t2=t1+ARS responder/non-responder (categorical); t2=t1+ARS change in percentage (continuous).

[5]Kruskal-Wallis was used because GLM normality assumption was not met.: t2-t1=ARS responder/non-responder (categorical).

[6]Spearman rank correlation was used because GLM normality assumption was not met: t2-t1=ARS change in percentage (continuous).

Supplementary table 5 Significant clusters (p_{FWE}<0.05) with their (sub-)peak coordinates for all contrasts of the stop-signal task (n=53)

	Hemisphere	MNI coordinates x, y, z (mm)	Cluster size	Peak *t*-value	p_{FWE} cluster
**StopSuccess>Go[1,2]**					
Middle Temporal Gyrus	Right	45, -66, 3	6511	13.04	<0.001
Middle Occipital Gyrus	Left	-48, -75, 3		12.38	
Superior Occipital Gyrus	Right	30, -69, 38		9.11	
Inferior Frontal Gyrus (orbitalis)	Left	-33, 27, -4	249	6.45	<0.001
Inferior Frontal Gyrus (triangularis)	Left	-33, 27, 6		6.30	
Insula	Left	-33, 18, -8		5.71	
Precentral	Left	-24, -6, 52	406	6.39	<0.001
Precentral / Inferior Frontal Gyrus (operculum)	Left	-45, 3, 27		5.85	
Middle Frontal Gyrus	Left	-33, 6, 66		4.93	
Thalamus	Right	6, -27, -1	230	6.19	<0.001
Thalamus	Left	-9, -27, -1		6.03	
Middle Cingular Gyrus	Right	6, 15, 45	148	6.04	<0.001
Supplementary Motor Area	Left	-9, 12, 48		4.88	
Middle Cingular Gyrus	Right	18, -30, 41	51	5.30	0.046
Precuneus	Right	9, -42, 55		3.43	
**Go>StopSuccess[1]**					
Postcentral Gyrus	Left	-42, -21, 55	424	10.79	<0.001
Precentral Gyrus	Right	51, -15, 55	485	10.39	<0.001
Cerebellum_6	Right	15, -57, -18	113	6.30	0.001
Vermis_6	Right	3, -66, -11		5.03	
Supplementary Motor Area	Left	-6, -6, 59	99	6.00	0.002
Putamen	Right	30, -3, -1	122	5.17	0.001
Insula	Right	36, -18, 10		4.72	
Cerebellum_4_5	Left	-18, -54, -18	67	4.90	0.016
Cerebellum_6	Left	-24, -48, -25		4.53	
**StopSuccess>StopFail[1]**					
Middle Cingular Gyrus	Left	-12, -36, 48	84	5.78	0.009
Superior Temporal Gyrus	Right	57, -21, 10	61	4.40	0.033
**StopFail>StopSuccess[1]**					
Inferior Frontal Gyrus (triangularis)	Left	-48, 15, -1	148	5.65	<0.001
Insula	Left	-36, 15, 3		4.98	
Inferior Frontal Gyrus (orbitalis)	Left	-36, 24, -8		3.51	
Anterior Cingular Gyrus	Right	9, 27, 27	216	5.32	<0.001
Anterior Cingular Gyrus	Left	-3, 30, 27		5.06	

MNI coordinates=Montreal Neurological Institute stereotaxic coordinates. p_{FWE}-value=family-wise-error-corrected p-value. Sub-peaks are indented.

[1]Maximally two additional sub-peaks are mentioned if their coordinates were in a different anatomical region or hemisphere.

[2]For the ROIs, the threshold for the StopSuccess>Go contrast was set to p_{FWE}<0.05 peak level to separate adjacent clusters. This led to the following additional regions not mentioned in this table: right insula/Inferior Frontal Gyrus-orbitalis (x=33, y=27, z=-4), left Middle Occipital Gyrus (x=-33, y=-72, z=27), two right precentral Gyrus clusters (x=45, y=3, z=48; x=27, y=-6, z=52), and left Superior Parietal Lobe (x=-24, y=-66, z=55).

Supplementary table 6 Comparison of mean beta weights of the stop-signal task ROIs at t2 *versus* t1, in relation to the relative ARS score decrease[1]

ROI (MNI coordinates x, y, z)	Responder[2] n=36		Non-responder[3] n=17		t1[4] (p-value)	ARS score decrease[4] (p-value)	Age[4] (p-value)	IQ[4] (p-value)	Medication[4] (p-value)	Continuous n=53		t1[5] (p-value)	ARS score decrease[5] (p-value)	Age[e] (p-value)	IQ[5] (p-value)	Medication[5] (p-value)
	t1 (SD)	t2 (SD)	t1 (SD)	t2 (SD)						t1 (SD)	t2 (SD)					
**StopSuccess>Go**																
Middle Temporal Gyrus	0.52	0.49	0.73	0.50	0.35	-0.02	-0.04	-0.003	-0.09	0.75	0.70	0.35	-0.0008	-0.03	-0.003	-0.11
Right (45, -66, 3)	(0.77)	(0.61)	(0.65)	(0.57)	(0.007)	(0.88)	(0.65)	(0.63)	(0.55)	(0.58)	(0.51)	(0.008)	(0.72)	(0.70)	(0.64)	(0.51)
Middle Occipital Gyrus	0.69	0.62	0.78	0.59	0.24	-0.05	0.12	-0.01	-0.26	0.24	-0.001	0.12	-0.009	-0.27	0.24	-0.001
Left (-48, -75, 3)	(0.56)	(0.53)	(0.59)	(0.33)	(0.05)	(0.72)	(0.17)	(0.12)	(0.08)	(0.053)	(0.58)	(0.15)	(0.12)	(0.07)	(0.053)	(0.58)
Middle Occipital Gyrus	0.43	0.57	0.53	0.62	0.30	-0.02	-0.12	-0.003	-0.14	0.46	0.59	0.30	-0.003	-0.12	-0.003	-0.14
Left (-33, -72, 27)	(0.51)	(0.68)	(0.58)	(0.56)	(0.07)	(0.91)	(0.27)	(0.70)	(0.47)	(0.53)	(0.64)	(0.07)	(0.92	(0.28	(0.70)	(0.48)
Superior Occipital Gyrus	0.41	0.47	0.49	0.67	0.21	0.06	-0.09	-0.0002	0.06	0.44	0.67	0.21	0.0003	-0.09	-0.0003	0.05
Right (30, -69, 38)	(0.58)	(0.75)	(0.48)	(0.38)	(0.19)	(0.74)	(0.35)	(0.98)	(0.73)	(0.47)	(0.53)	(0.20)	(0.90)	(0.38)	(0.96)	(0.78)
Frontal Inferior Orbital	0.44	0.47	0.44	0.73	-0.06	-0.22	0.07	0.01	0.14	-0.06	-0.003	0.07	0.009	0.13	-0.06	-0.003
Left (-33, 27, -4)	(0.80)	(0.71)	(0.68)	(0.77)	(0.69)	(0.34)	(0.60)	(0.33)	(0.56)	(0.64	(0.43	(0.60)	(0.30	(0.57)	(0.64	(0.43)
Insula	0.72	0.69	0.82	0.73	-0.09	-0.21	-0.02	-0.01	-0.22	0.55	0.51	-0.10	-0.003	-0.02	-0.008	-0.24
Right (33, 27, -4)	(0.59)	(0.60)	(0.58)	(0.24)	(0.50)	(0.38)	(0.87)	(0.34)	(0.32)	(0.79)	(0.74)	(0.45)	(0.41)	(0.91)	(0.38)	(0.31)
Precentral	0.39	0.36	0.32	0.48	0.19	-0.16	0.12	0.01	-0.04	0.37	0.40	0.17	-0.002	0.12	0.006	-0.04
Left (-24, -6, 52)	(0.44)	(0.61)	(0.51)	(0.41)	(0.26)	(0.36)	(0.23)	(0.35)	(0.84)	(0.46)	(0.55)	(0.34)	(0.47)	(0.23)	(0.33)	(0.82)
Precentral	0.59	0.31	0.42	0.50	-0.06	-0.11	-0.02	-0.01	0.31	0.54	0.37	-0.07	-0.002	-0.02	-0.008	0.30
Left (-45, 3, 27)	(1.13)	(0.75)	(0.78)	(0.58)	(0.51)	(0.63)	(0.84)	(0.31)	(0.16)	(1.03)	(0.70)	(0.48)	(0.65)	(0.86)	(0.33)	(0.18)
Precentral	0.42	0.68	0.42	0.63	-0.07	-0.36	0.12	0.02	0.27	-0.09	-0.006	0.13	0.02	0.22	-0.09	-0.006
Right (27, -6, 52)	(0.60)	(0.59)	(0.55)	(0.69)	(0.66)	(0.07)	(0.29)	(0.04)	(0.16)	(0.55)	(0.04)	(0.22)	(0.02)	(0.27)	(0.55)	(0.04)
Precentral	0.69	0.57	0.48	1.47	-0.04	0.02	-0.05	0.0003	0.08	-0.05	0.001	-0.04	0.0002	0.05	-0.05	0.001
Right (45, 3, 48)	(1.36)	(1.33)	(1.22)	(1.73)	(0.71)	(0.92)	(0.63)	(0.97)	(0.68)	(0.65)	(0.71)	(0.73)	(0.97)	(0.81)	(0.65)	(0.71)
Thalamus	0.28	0.44	0.41	0.66	-0.36	-0.97	0.05	-0.02	-0.43	0.62	0.86	-0.34	-0.01	0.05	-0.02	-0.44
Right (6, -27, -1)	(0.79)	(0.77)	(0.58)	(0.69)	(0.03)	(0.03)	(0.84)	(0.16)	(0.35)	(1.31)	(1.51)	(0.04)	(0.07)	(0.84)	(0.21)	(0.35)
Thalamus	0.48	0.54	0.18	0.88	-0.28	-0.40	-0.02	-0.02	-0.47	-0.28	-0.005	-0.02	-0.02	-0.47	-0.28	-0.005
Left (-9, -27, -1)	(1.00)	(0.99)	(0.59)	(0.82)	(0.06)	(0.17)	(0.91)	(0.12)	(0.10)	(0.07)	(0.28)	(0.91)	(0.15)	(0.11)	(0.07)	(0.28)
Middle Cingular Gyrus	0.03	0.44	0.44	-0.32	-0.01	-0.17	0.08	0.01	0.23	0.32	0.51	-0.02	-0.003	0.09	0.01	0.21
Right (6, 15, 45)	(0.95)	(1.00)	(0.84)	(0.90)	(0.95)	(0.47)	(0.55)	(0.27)	(0.32)	(0.73)	(0.75)	(0.89)	(0.43)	(0.51)	(0.26)	(0.39)
Middle Cingular Gyrus	0.25	0.21	0.14	0.40	-0.10	-0.22	0.04	0.01	-0.05	0.22	0.25	-0.11	-0.005	0.06	0.008	-0.11
Right (18, -30, 41)	(0.49)	(0.63)	(0.52)	(0.33)	(0.44)	(0.11)	(0.60)	(0.16)	(0.68)	(0.49)	(0.43)	(0.36)	(0.02)	(0.40)	(0.12)	(0.39)
Superior Parietal lobe	0.51	0.43	0.46	0.36	0.16	-0.04	0.05	-0.01	-0.33	0.49	0.41	0.16	-0.001	0.03	-0.004	-0.29
Left (-24, -66, 55)	(0.55)	(0.63)	(0.56)	(0.56)	(0.32)	(0.85)	(0.62)	(0.55)	(0.08)	(0.55)	(0.60)	(0.31)	(0.66)	(0.75)	(0.55)	(0.14)

(continued on next page)

Supplementary table 6 (continued)

	Responder[2] n=36		Non-responder[3] n=17			ARS				Continuous n=53			ARS			
**ROI (MNI coordinates x, y, z)**	**t1 (SD)**	**t2 (SD)**	**t1 (SD)**	**t2 (SD)**	**t1[4] (p-value)**	**score decrease[4] (p-value)**	**Age[4] (p-value)**	**IQ[4] (p-value)**	**Medication[4] (p-value)**	**t1 (SD)**	**t2 (SD)**	**t1[5] (p-value)**	**score decrease[5] (p-value)**	**Age[5] (p-value)**	**IQ[5] (p-value)**	**Medication[5] (p-value)**
**Go>StopSuccess**																
Postcentral	-0.77	-0.77	-0.60	-0.75	0.15	0.16	-0.11	-0.005	0.55	-0.72	-0.77	0.17	0.004	-0.12	-0.005	0.60
Left (-42, -21, 55)	(0.85)	(0.99)	(0.72)	(0.70)	(0.33)	(0.56)	(0.51)	(0.67)	(0.051)	(0.81)	(0.90)	(0.28)	(0.38)	(0.44)	(0.64)	(0.04)
Precentral	-0.75	-0.88	-0.77	-0.74	0.19	0.01	-0.04	-0.001	0.56	-0.75	-0.83	0.19	-0.001	-0.03	-0.001	0.53
Right (51, -15, 55)	(1.00)	(0.87)	(0.72)	(0.89)	(0.16)	(0.98)	(0.77)	(0.89)	(0.04)	(0.91)	(0.87)	(0.16)	(0.77)	(0.84)	(0.89)	(0.06)
Cerebellum	-0.46	-0.45	-0.14	-0.19	-0.16	-0.26	-0.02	-0.01	0.22	-0.36	-0.36	-0.16	-0.004	-0.007	-0.01	0.20
Right (15, -57, -18)	(0.58)	(0.67)	(0.54)	(0.74)	(0.37)	(0.24)	(0.90)	(0.09)	(0.31)	(0.58)	(0.69)	(0.37)	(0.24)	(0.95)	(0.10)	(0.38)
Supplementary Motor Area	-0.43	-0.42	-0.37	-0.36	0.07	0.02	0.07	0.01	0.26	-0.41	-0.40	0.07	0.001	0.07	0.01	0.24
(-6, -6, 59)	(0.65)	(0.81)	(0.76)	(0.71)	(0.66)	(0.95)	(0.64)	(0.21)	(0.28)	(0.68)	(0.77)	(0.67)	(0.85)	(0.60)	(0.21)	(0.33)
Putamen	-0.36	-0.47	-0.19	-0.23	0.06	-0.24	0.05	-0.01	0.03	-0.31	-0.39	0.05	-0.003	0.06	-0.01	0.02
Right (30, -3, -1)	(0.56)	(0.50)	(0.72)	(0.58)	(0.63)	(0.15)	(0.57)	(0.10)	(0.84)	(0.61)	(0.53)	(0.70)	(0.18)	(0.55)	(0.13)	(0.90)
Cerebellum	-0.45	-0.56	-0.07	-0.06	-0.18	-0.45	-0.13	-0.01	0.38	-0.33	-0.40	-0.19	-0.01	-0.12	-0.01	0.34
Left (-18, -54, -18)	(0.58)	(0.74)	(0.64)	(0.91)	(0.38)	(0.08)	(0.36)	(0.15)	(0.13)	(0.62)	(0.82)	(0.20)	(0.08)	(0.41)	(0.18)	(0.18)
**StopSuccess>StopFail**																
Middle Cingular Gyrus	0.27	0.33	0.20	0.27	-0.24	0.18	-0.16	0.01	0.18	-0.22	0.001	-0.16	0.01	0.16	-0.22	0.001
Left (-12, -36, 48)	(0.67)	(0.53)	(0.30)	(0.50)	(0.06)	(0.23)	(0.07)	(0.02)	(0.23)	(0.08)	(0.61)	(0.098)	(0.03)	(0.31)	(0.08)	(0.61)
Superior Temporal Gyrus	0.09	0.31	0.40	0.51	-0.01	-0.16	-0.06	-0.01	0.13	0.01	-0.006	-0.07	-0.01	0.16	0.01	-0.006
Right (57, -21, 10)	(0.66)	(0.63)	(0.50)	(0.65)	(0.96)	(0.45)	(0.59)	(0.20)	(0.51)	(0.95)	(0.85)	(0.53)	(0.22)	(0.44)	(0.95)	(0.85)
**StopFail>StopSuccess**																
Inferior Frontal Gyrus (triangularis)	-0.52	-0.29	-0.37	-0.12	-0.05	-0.15	-0.04	-0.001	0.05	-0.04	-0.001	-0.04	-0.0008	0.06	-0.04	-0.001
Left (-48, 15, -1)	(0.91)	(0.54)	(0.65)	(0.51)	(0.63)	(0.39)	(0.73)	(0.86)	(0.75)	(0.65)	(0.58)	(0.71)	(0.91)	(0.72)	(0.65)	(0.58)
Anterior Cingular Gyrus	-0.31	-0.35	-0.29	-0.27	0.18	-0.02	-0.02	-0.002	0.20	0.18	-0.0001	-0.02	-0.002	0.21	0.18	-0.0001
Right (9, 27, 27)	(0.56)	(0.62)	(0.45)	(0.52)	(0.27)	(0.89)	(0.86)	(0.74)	(0.29)	(0.27)	(0.98)	(0.84)	(0.75)	(0.28)	(0.27)	(0.98)

ARS=Attention-deficit hyperactivity disorder (ADHD) Rating Scale. df=degrees of freedom. FFD=few-foods diet. IQ=intelligence quotient. MNI coordinates=Montreal Neurological Institute stereotaxic coordinates. ROI=region of interest (n=25). SD=standard deviation. t1=before start FFD. t2=at the end of the FFD.

[1]ARS score decrease=100x(t1-t2)/t1 ARS score.

[2]Responders showed ≥40% ARS score decrease at t2 compared to t1.

[3]Non-responders showed <40% ARS score decrease at t2 compared to t1.

[4]GLM: t2=t1+ARS score decrease (responder/non-responder)+age+IQ+previous use of medication (yes/no). The values represent: coefficient of the predictor variable (p-value). P-values <0.002 are considered significant (0.05/25); p-values <0.004 (0.1/25) are considered a trend.

[5]GLM: t2=t1+ARS score decrease in percentage+age+IQ+previous use of medication (yes/no). The values represent: coefficient of the predictor variable (p-value). P-values <0.002 are considered significant (0.05/25); p-values <0.004 (0.1/25) are considered a trend.

Supplementary Figures

a b

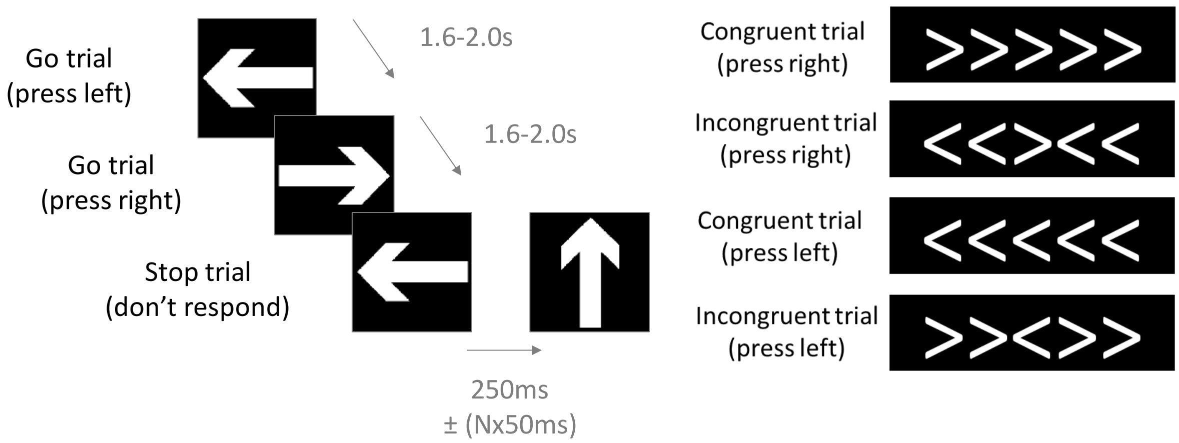

Supplementary figure 1 Stop-signal task and Flanker task design. (a) Stop-signal task design (1), with 80% Go trials and 20% Stop trials. (b) Flanker task design (2), with 50% congruent trials and 50% incongruent trials. The congruent and incongruent targets appeared pseudo-randomly intermixed on the screen with an inter-trial interval of 2-6 seconds and remained on the screen for 750 ms.

1. Rubia, K., Smith, A. B., Brammer, M. J. & Taylor, E. Right inferior prefrontal cortex mediates response inhibition while mesial prefrontal cortex is responsible for error detection. NeuroImage 20, 351-358 (2003).
2. Eriksen, B. A. & Eriksen, C. W. Effects of noise letters upon the identification of a target letter in a nonsearch task. Perception & Psychophysics 16, 143-149 (1974).

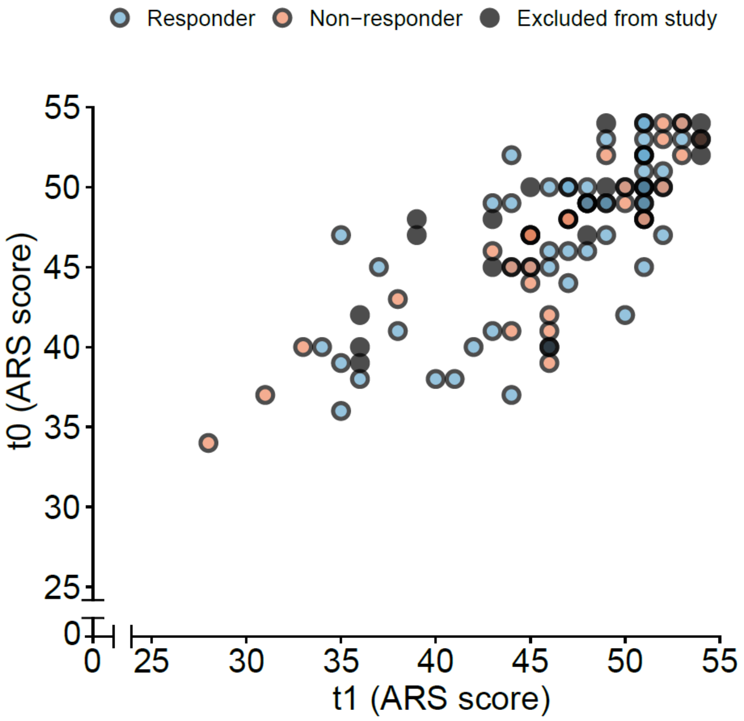

Supplementary figure 2 ARS scores at t0 and t1 (n=100). Blue and red dots represent responders (≥40% ARS score decrease at t2 compared to t1; n=50) and non-responders (<40% ARS score decrease at t2 compared to t1; n=29) respectively. Black dots represent children that were excluded before or at t2 (n=21). Minimum ARS score=0, maximum score=54. ARS=Attention-deficit hyperactivity disorder (ADHD) Rating Scale. FFD=few-foods diet. t0=screening. t1=before start FFD. t2=at the end of the FFD.

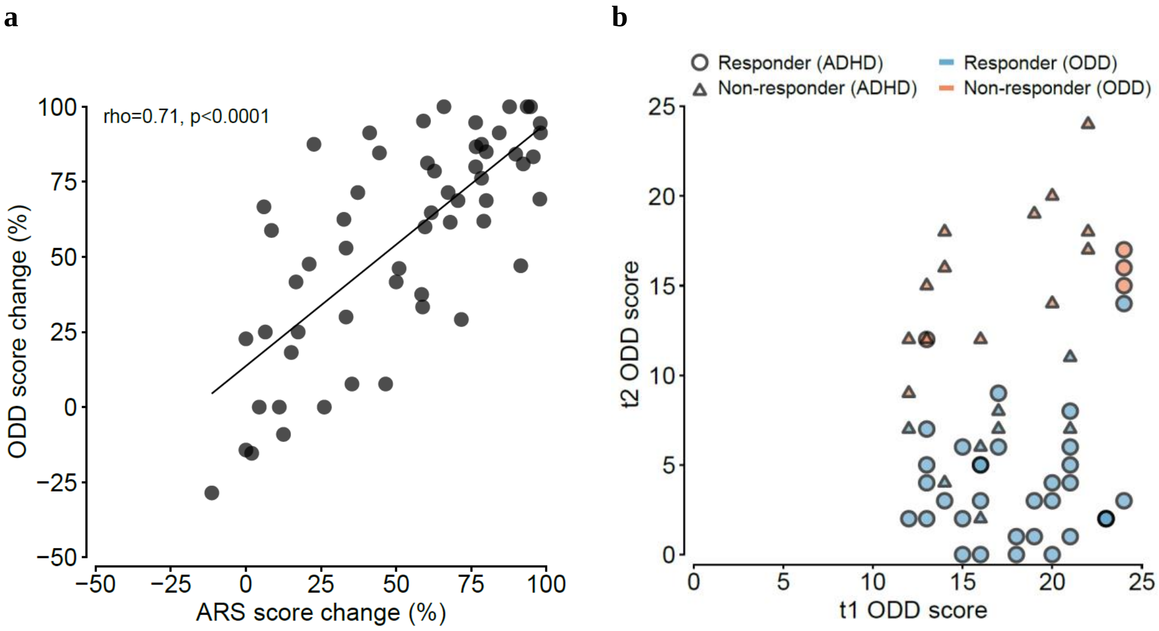

Supplementary figure 3 Correlation between ADHD and ODD symptom scores following an FFD. (a) Correlation between ARS score change and ODD score change (n=57; Spearman-rank test). (b) ODD scores (minimum score=0, maximum score=24) at t1 and t2 (n=57). Blue colour represents ODD responders (≥40% ODD score decrease at t2 compared to t1; n=40) and red colour represents ODD non-responders (<40% ODD score decrease at t2 compared to t1; n=17). Circles represent ADHD responders (≥40% ADHD score decrease at t2 compared to t1) and triangles represent ADHD non-responders (<40% ADHD score decrease at t2 compared to t1).

ODD+ADHD responder: n=32

ODD+ADHD nonresponder: n=13

ODD responder + ADHD nonresponder: n=8

ODD nonresponder + ADHD responder: n=4

ADHD= Attention-deficit hyperactivity disorder. FFD=few-foods diet. t1 ODD=Oppositional defiant disorder. t1=before start FFD. t2=at the end of the FFD.
